# Molecular Evolutionary Pathways toward Two Successful Community-Associated but Multidrug-Resistant ST59 Methicillin-Resistant *Staphylococcus aureus* Lineages in Taiwan: Dynamic Modes of Mobile Genetic Element Salvages

**DOI:** 10.1371/journal.pone.0162526

**Published:** 2016-09-08

**Authors:** Wei-Chun Hung, Tsai-Wen Wan, Yu-Chia Kuo, Tatsuo Yamamoto, Jui-Chang Tsai, Yu-Tzu Lin, Po-Ren Hsueh, Lee-Jene Teng

**Affiliations:** 1 Department of Microbiology and Immunology, Kaohsiung Medical University, Kaohsiung, Taiwan; 2 Department of Clinical Laboratory Sciences and Medical Biotechnology, National Taiwan University College of Medicine, Taipei, Taiwan; 3 Department of Epidemiology, Genomics, and Evolution, International Medical Education and Research Center, Niigata, Japan; 4 Institute of Medical Device and Imaging, National Taiwan University College of Medicine, Taipei, Taiwan; 5 Division of Neurosurgery, Department of Surgery, National Taiwan University Hospital, Taipei, Taiwan; 6 Department of Laboratory Medicine, National Taiwan University Hospital, Taipei, Taiwan; Rockefeller University, UNITED STATES

## Abstract

Clonal complex 59 (CC59) S*taphylococcus aureus* in Taiwan includes both methicillin-susceptible *S*. *aureus* (MSSA) and methicillin-resistant *S*. *aureus* (MRSA). As the most prominent community-associated MRSA (CA-MRSA) in Taiwan, CC59 has two major clones characterized as PVL-negative SCC*mec* IV (carrying the staphylococcal cassette chromosome *mec* IV but Panton-Valentine leukocidin-negative) and PVL-positive SCC*mec* V (5C2&5). We investigated the drug resistance, phylogeny and the distribution and sequence variation of SCC*mec*, staphylococcal bacteriophage φSA3, genomic island νSaβ and MES (an enterococcal mobile genetic element conferring multidrug resistance) in 195 CC59 *S*. *aureus*. Sequencing and PCR mapping revealed that all of the CC59/SCC*mec* V (5C2&5) MRSA strains had acquired MES_PM1_ or its segregants, and obtained a φSA3-related fragment in νSaβ. In contrast, MES_6272-2_ and MES_4578_, which showed gentamicin resistance that was not encoded by MES_PM1_, were dominant in SCC*mec* IVg MRSA. Translocation of a whole φSA3 into νSaβ instead of only a φSA3-related fragment was common in SCC*mec* IVg MRSA. However, the non-subtype-g SCC*mec* IV MRSA (SCC*mec* IVa is the major) still carried MES and νSaβ structures similar to those in SCC*mec* V (5C2&5) MRSA. A minimum spanning tree constructed by multiple-locus variable-number tandem repeat analysis revealed that SCC*mec* IVg MRSA and SCC*mec* V (5C2&5) MRSA grouped respectively in two major clades. The CC59 MSSA was equally distributed among the two clades, while the non-subtype-g SCC*mec* IV MRSA mostly clustered with SCC*mec* V (5C2&5) MRSA. Our findings strongly suggest that CC59 MSSA acquired divergent mobile genetic elements and evolved to SCC*mec* IVg MRSA and SCC*mec* V (5C2&5) MRSA/non-subtype-g SCC*mec* IV MRSA independently. The evolutionary history of CC59 *S*. *aureus* explains how mobile genetic elements increase the antimicrobial resistance and virulence and contribute to the success of CA-MRSA in Taiwan.

## Introduction

*Staphylococcus aureus* poses significant public health challenges worldwide. It is well known that early acquisition of the staphylococcal chromosome cassette (SCC) *mec* in 1961 led to methicillin-resistant *S*. *aureus* (MRSA) [1, 2]. Since then, MRSA has imposed a heavy burden in healthcare environments, where it is also known as healthcare-associated MRSA (HA-MRSA) [1, 2]. In the late 1990s, another genetically distinct MRSA, designated community-associated MRSA (CA-MRSA), emerged in the community, causing skin and soft tissue infections (SSTIs) and severe clinical disease, such as necrotizing pneumonia, in children and young adults without antecedent healthcare exposure [2–5]. CA-MRSA is usually susceptible to non-β-lactam antibiotics, in contrast to HA-MRSA [2, 4, 6, 7]. However, accumulation of increased drug resistance and increasing incidence in healthcare facilities has been noted in some CA-MRSA strains, such as the USA300 clone, which emerged in the United States [8].

Currently, five major CA-MRSA clones with sequence type (ST) 1, ST8, ST30, ST59 and ST80 are distributed in specific geographic areas [9, 10]. In Taiwan, the dominant CA-MRSA clone is clonal complex (CC) 59 MRSA, which is responsible for 56% of pediatric CA-MRSA infections [11]. CC59 MRSA is composed of two genotypes: (i) the Asian-Pacific clone, characterized as Panton-Valentine leukocidin (PVL)-negative ST59/SCC*mec* IV MRSA, which is prevalent among colonizing isolates and in hospitals; and (ii) the Taiwan clone, characterized as PVL-positive ST59/SCC*mec* V (5C2&5) MRSA, which is dominant in the clinical isolates [10, 12]. The Taiwan clone possesses a novel SCC*mec* with two distinct *ccrC* genes (*ccrC1* allele 2 and *ccrC1* allele 8) [13]. The novel SCC*mec* was firstly named SCC*mec* V_T_ [14] and later tentatively designated as SCC*mec* VII [15]. Finally, it was reclassified as SCC*mec*V (5C2&5) by the International Working Group on the Classification of Staphylococcal Cassette Chromosome Elements [13].The CC59 MRSA strain has also been reported in Singapore [16], Hong Kong [17], Japan [18, 19], western Australia [20] and Europe [21].

The Taiwan clone possesses several features that are rarely found in other CA-MRSA: (i) resistance to at least four classes of non-β-lactam antimicrobials since it was first isolated in 1997 [14, 22]; (ii) acquisition of MES_PM1_, which originated in enterococci, encoding resistance to erythromycin, clindamycin, kanamycin, streptomycin and chloramphenicol [23]; (iii) truncation of type I restriction-modification (R-M) system genes (*hsdS* and *hsdM*) in genomic island νSaβ [23]; and (iv) retention of the immune evasion cluster (IEC) type C genes *chp* (encoding CHIPS, chemotaxis inhibitory protein) and *scn* (encoding SCIN, staphylococcal complement inhibitor) and loss of the Hlb-converting prophage φSA3 [23, 24]. *S*. *aureus* strains carry the IEC genes *chp*, *sak* (encoding SAK, staphylokinase), *scn*, *sea* (encoding SEA, staphylococcal enterotoxin A) and *sep* (encoding SEP, staphylococcal enterotoxin P) with different combinations [25]. The IEC genes usually cluster on the 3ˈ-end of φSA3 and integrate into *hlb* [25]. However, the Taiwan clone harbors IEC type C in the νSaβ rather than in φSa3. The acquisition disrupts *hsdS* and *hsdM*, which leads to defects in the type I R-M system that is known to block horizontal gene transfer [26] and thus may partly contribute to the introduction of foreign DNA (e.g., MES_PM1_) in the Taiwan clone [23].

In contrast, the Asian-Pacific clone carries an intact φSA3 with IEC type G, which carries *sak* (encoding SAK, staphylokinase), *sep* (encoding SEP, staphylococcal enterotoxin P) and *scn* based on microarray analysis [24]. The Asian-Pacific clone is also resistant to various non-β-lactam antibiotics, such as erythromycin, clindamycin, kanamycin, streptomycin and gentamicin, although more isolates with resistance to gentamicin and fewer isolates with resistance to streptomycin were noted [27, 28]. However, the genetic characteristics of IEC and multidrug resistance genes remain unknown.

In Taiwan, CC59 *S*. *aureus* also comprised 8.8% and 8% of the methicillin-susceptible *S*. *aureus* (MSSA) infection and nasal carriage isolates, respectively [29]. An enterococcal IS*1216V*-mediated multidrug resistance structure nearly identical to MES_PM1_ has been reported in the PVL-positive ST59 MSSA strain KS1 [19]. Moreover, PVL-positive ST59 MSSA displayed high similarity in PFGE pattern (>80%) and had an identical genetic profile (*spa*-CC c2:441/437 and *agr* group I) to PVL-positive ST59/SCC*mec* V (5C2&5) MRSA [30].

CC59 *S*. *aureus* is the only genotype in Taiwan that may be either MRSA or MSSA [30, 31]. The co-existence of CC59 MSSA, SCC*mec* IV MRSA and SCC*mec* V (5C2&5) MRSA with unique features in Taiwan may represent a novel evolutionary history. Therefore, we collected 195 CC59 *S*. *aureus* strains isolated from northern Taiwan in 2000, 2005, 2010 and 2011 and investigated the distribution of mobile genetic elements, including SCC*mec*, νSaβ, φSA3 and MES-related elements, within these isolates. The phylogenetic relationships of the CC59 *S*. *aureus* strains were determined by multiple-locus variable-number tandem repeat analysis (MLVA) [32].

## Materials and Methods

### Bacterial isolates

A total of 195 CC59 *S*. *aureus* isolates obtained from the National Taiwan University Hospital in 2000, 2005, 2010 and 2011 were investigated in this study (S1 Table); 176 strains were collected from blood culture, and 19 strains were isolated from various specimens (skin and soft tissue, sputum, abscess, etc.) that were characterized as CA-MRSA in our previous study [33]. All isolates were identified as CC59 *S*. *aureus* through *spa* type identification using the RIDOM *spa* server database [34]. The sequence types of 25 isolates were further confirmed by multilocus sequence typing (MLST) [35]. Three ST59 CA-MRSA strains were used as reference strains: strain PM1 (SCC*mec* V (5C2&5)) was characterized in our previous study [23], and TSGH17 (SCC*mec* V (5C2&5)) from Taiwan [14] and USA1000 (SCC*mec* IVa) from the United States [36] were kindly provided by C. C. Wang and by L. K. McDougal and L. C. McDonald, respectively.

### Antimicrobial susceptibility testing

Antimicrobial susceptibility testing was performed by the agar dilution method according to the CLSI 2015 guidelines [37] for erythromycin, kanamycin, streptomycin, gentamicin and chloramphenicol. *S*. *aureus* ATCC 29213 was used as the reference strain.

### SCC*mec* typing

Characterization of SCC*mec* and subtyping of SCC*mec* IV elements were performed by PCR as previously described [33, 38, 39].

### Whole genome sequencing

The genome sequence of the multidrug-resistant ST59/SCC*mec* IVg MRSA strain 4578 was analyzed with an Illumina Genome Analyzer (Illumina, San Diego, CA). Sequencing yielded 1,158,721,894 bp of raw sequence in 11,472,494 reads, which was approximately 413 times the size of the genome. The 913,070 reads were mapped to the genome of ST59/SCC*mec* V (5C2&5) MRSA strain M013 (accession number CP003166). Contigs were obtained using de novo assembly with an algorithm of Velvet [40]. The gaps between contigs related to mobile genetic elements were filled by PCR and sequencing. Open reading frames were analyzed using the DNAman software package (Version 6) (Lynnon Biosoft, Quebec, Canada).

### Detection of resistance determinants and virulence genes

The presence of resistance determinants (*ermB*, *aph(3')-IIIa*, *aadE*, *aacA-aphD* and *cat*) and virulence genes (*luk*_*PV*_*SF*, *hlb*, *chp*, *sak*, *scn*, *sea* and *sep*) was determined by PCR as previously described [25, 27].

### Detection of MES, νSaβ and φSA3 structures

The MES structure was mapped by PCR using primer sets a to d. The νSaβ structure was mapped using primer sets e to f. φSA3 integrated within *hlb* was shown using primer set i and j. All of the primer sets are listed in S2 Table, and the positions of the primers are indicated in S1 Fig. Combing the PCR mapping results and the profiles of resistance determinants or virulence genes, the MES, νSaβ and φSA3 structures were determined.

### Multiple-locus variable-number tandem repeat analysis (MLVA)

The MLVA-16_orsay_ PCR with 16 loci was performed as previously described by Sobral et al. [32]. The PCR products were resolved by gel electrophoresis in 1.2 or 3% agarose-0.5× Tris-borate-EDTA. PCR products showing different sizes for each loci were chosen for sequencing to determine the exact numbers of tandem repeats. The MLVA codes were provided in the order corresponding to the genome position in reference strain Mu50 (accession number NC_002758): Sa0122, Sa0266, Sa0311, Sa0704, Sa1132, Sa1194, Sa1291, Sa1729, Sa1866, Sa2039, Sa0387, Sa0550, Sa0684, Sa0964, Sa1097 and Sa2511. The MLVA profile data were imported into BioNumerics (Applied-Maths, Sint-Martens-Latem, Belgium) to construct the minimum spanning tree.

### Selection for loss of antibiotic resistance in strain PM1

Loss of antibiotic resistance to erythromycin, kanamycin, streptomycin and chloramphenicol in the ST59/SCC*mec*V MRSA strain PM1 was tested. After growing overnight on Mueller Hinton II agar (Difco) supplemented with 32 ug/ml erythromycin and 32 ug/ml chloramphenicol, the PM1 was suspended into fresh Mueller Hinton broth (Difco) without antibiotics and inoculated in 37°C for 24 h. The growing cultures were then diluted and spread on Mueller Hinton II agar plates. The resulting colonies were picked and subcultured onto plates containing erythromycin (32 ug/ml) or chloramphenicol (32 ug/ml). Of the 1920 colonies tested, five (PM1-1 to PM1-5) failed to appear on the appropriate antibiotic plate.

### Pulsed-field gel electrophoresis (PFGE)

Pulsed-field gel electrophoresis (PFGE) was performed as previously described [41]. In brief, The DNAs of PM1 and PM1-1 to PM1-5 were digested with SmaI (New England BioLabs, Ipswich, MA) and then were separated using a CHEF-DRIII apparatus (Bio-Rad Laboratories). PFGE was carried out at 200 V and 12°C for 20 h with the pulse times ranging from 5 to 60 s.

### Southern blot

The DNAs digested with SmaI and separated by the PFGE were transferred to nylon membranes (Amersham Hybond™-N; GE Healthcare) by vacuum blotting. The blotted membranes were prehybridized overnight in DIG Easy Hyb (Roche) at 42°C. The digoxigenin (DIG)-labeled probes specific to IS*1216V*, *ermB* and *cat* were prepared using DIG PCR Probe Synthesis Kit (Roche). The membrane was hybridized with the specific probe overnight in DIG Easy Hyb (Roche) at 42°C. The detection of hybridization was performed with an anti-DIG antibody conjugated to alkaline phosphatase, and CSPD (Roche) was used as a substrate according to the manufacturer’s instructions.

### Nucleotide sequences

The nucleotide sequences of SCC*mec* IVg, νSaβ (including φSA3_4578_) and MES_4578_ in strain 4578, MES_6272-2_ in strain 6272–2, MES_2250_ in strain 2250 and νSaβ in strain 187–4 have been deposited in the GenBank database under accession numbers LC125348 to LC125353.

### Statistics

The incidences of drug resistance, virulence genes and IEC types among SCC*mec* IV MRSA, SCC*mec* V (5C2&5) MRSA and MSSA were compared using Fisher's exact test. Statistical significance was defined as a *P* value of <0.05.

## Results

### Antimicrobial susceptibility and virulence gene distribution among the CC59 *S*. *aureus* strains

A total of 195 CC59 *S*. *aureus* strains were included in this study (S1 Table). The non-β-lactam antibiograms and distribution of virulence genes *luk*_*PV*_*SF*, *hlb* and IEC of the CC59 *S*. *aureus* strains are summarized in Table 1. Resistance to erythromycin, kanamycin and chloramphenicol was evenly distributed among the CC59 *S*. *aureus* strains. However, gentamicin resistance was predominantly found in SCC*mec* IV MRSA, while streptomycin resistance was mainly present in SCC*mec* V (5C2&5) MRSA and MSSA (Fisher’s exact test, *P* value < 0.05). Resistance to both gentamicin and streptomycin was found in only one SCC*mec* IV MRSA strain, two SCC*mec* V (5C2&5) MRSA strains and one MSSA strain (S3 Table). Moreover, strains resistant to gentamicin or streptomycin were always simultaneously resistant to both erythromycin and kanamycin (S3 Table).

**Table 1:** Drug resistance to non-β-lactams and presence of virulence genes in CC59 *S*. *aureus* strains.

	No. (%) of strains		
	SCC*mec* IV (n = 91)	SCC*mec* V (5C2&5) (n = 74)	MSSA (n = 30)
Drug resistance rate (%)			
Erythromycin	85 (93.4)	66 (89.2)	27 (90.0)
Kanamycin	80 (87.9)	66 (89.2)	27 (90.0)
Streptomycin ^a^	20 (22.0)	66 (89.2)	25 (83.3)
Gentamicin ^a^	58 (63.7)	2 (2.7)	3 (10.0)
Chloramphenicol	60 (65.9)	46 (62.2)	16 (53.3)
Susceptible to above antibiotics	3 (3.3)	6 (8.0)	3 (10.0)
Presence of virulence genes (%)			
*luk*_*PV*_*SF* ^b^	12 (13.2)	71 (95.9)	15 (50.0)
*hlb* ^c^	80 (87.9)	74 (100)	30 (100)
Immune evasion cluster (IEC)			
Type B: *sak*, *chp*, *scn* ^c^	13 (14.3)	0 (0)	1 (3.3)
Type C: *chp*, *scn* ^b^	8 (8.8)	74 (100)	25 (83.3)
Type D: *sea*, *sak*, *scn*	3 (3.3)	0 (0)	0 (0)
Type G: *sep*, *sak*, *scn* ^b^	63 (69.2)	0 (0)	4 (13.3)
No IEC	4 (4.4)	0 (0)	0 (0)

^a^ Statistically significant difference between SCC*mec* IV MRSA/SCC*mec* V (5C2&5) MRSA and between SCC*mec* IV MRSA/MSSA.

^b^ Statistically significant difference among SCC*mec* IV MRSA, SCC*mec* V (5C2&5) MRSA and MSSA.

^c^ Statistically significant difference between SCC*mec* IV MRSA/SCC*mec* V (5C2&5) MRSA.

Of the virulence genes, the *luk*_*PV*_*SF* gene (encoding PVL) was more prevalent in SCC*mec* V (5C2&5) MRSA compared with SCC*mec* IV MRSA or MSSA. The *hlb* gene, which is usually truncated due to φSA3 integration [23], was intact in all CC59 *S*. *aureus* isolates except 11 SCC*mec* IV MRSA strains. However, the IEC, which is usually carried by φSA3 [25], was still present in all CC59 *S*. *aureus* strains except 4 SCC*mec* IV MRSA strains. Moreover, the distribution of major IEC types was different (type G in SCC*mec* IV MRSA vs. type C in SCC*mec* V (5C2&5) MRSA and MSSA).

### Comparative genomics of the Asian-Pacific clone and the Taiwan clone

**Fig 1:**
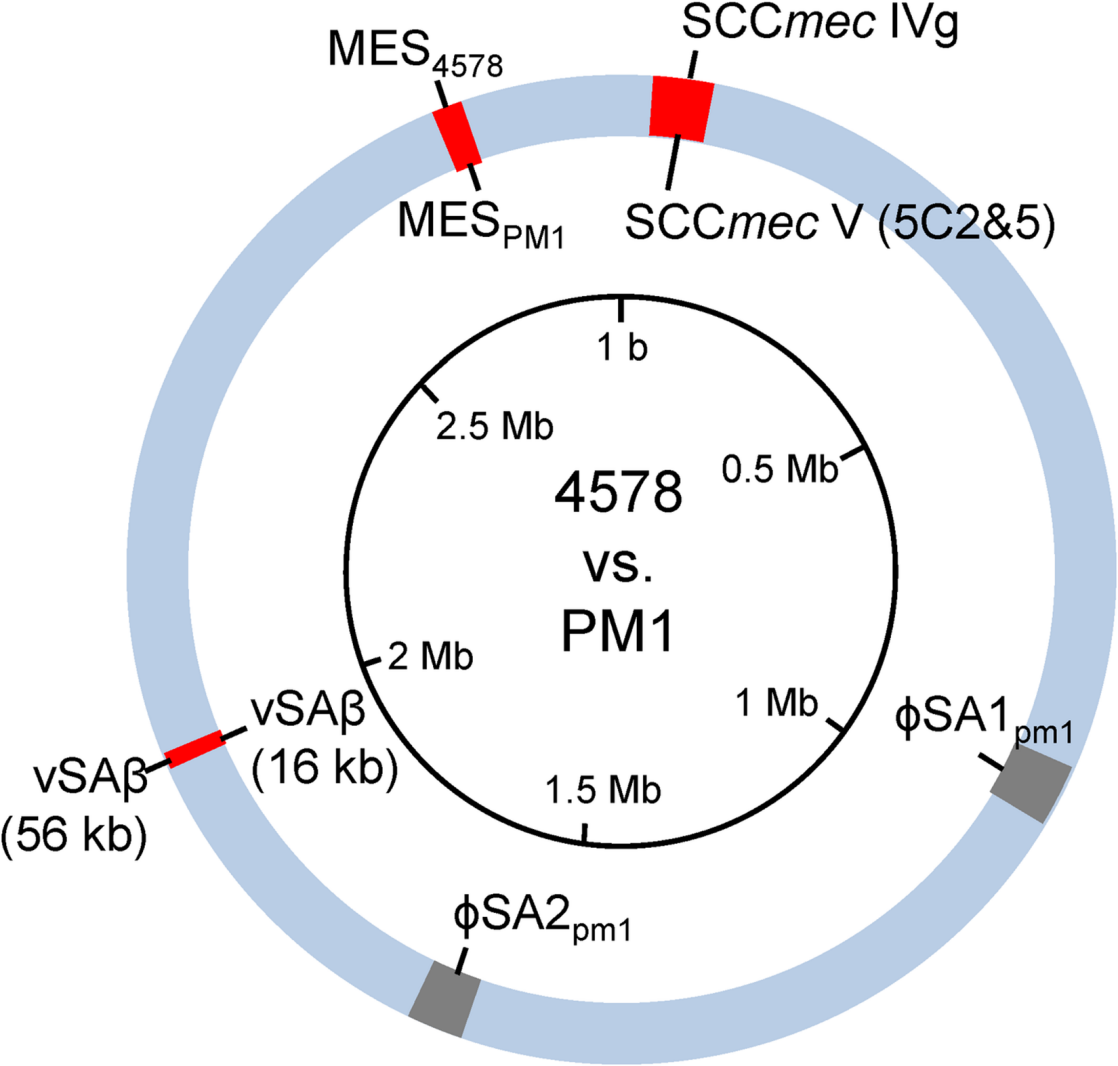
Comparative genomics of 4578 (ST59/SCC*mec* IVg MRSA) and PM1 (ST59/SCC*mec* V (5C2&5) MRSA). Information on the 4578 and PM1 genomes is presented outside and inside the genome circle, respectively. Colored regions: blue, conserved region; gray, deletion in strain 4578; red, distinct mobile genetic elements.

Because the Asian-Pacific clone showed distinct differences in gentamicin/streptomycin resistance and IEC types (Table 1), we were curious if any novel genetic organization was present, similar to MES_PM1_ and νSAβ in the Taiwan clone [23]. Therefore, the multidrug-resistant SCC*mec* IV MRSA strain 4578 was chosen for whole genome sequencing, and the results were compared with those from the SCC*mec* V (5C2&5) MRSA strain PM1 that we previously analyzed [23]. As summarized in Fig 1, two large deletions and three mobile genetic elements with marked divergence were identified: (i) the bacteriophages φSA1_pm1_ and φSA2_pm1_ (carrying the *luk*_*PV*_*SF* gene) were absent in SCC*mec* IV MRSA strain 4578; and (ii) three mobile genetic elements, SCC*mec* IVg, MES_4578_ and a 56-kb νSAβ, were unique in SCC*mec* IV MRSA strain 4578. The structures of MES_4578_ and νSAβ are discussed below.

### Structures of multidrug resistance elements

**Fig 2:**
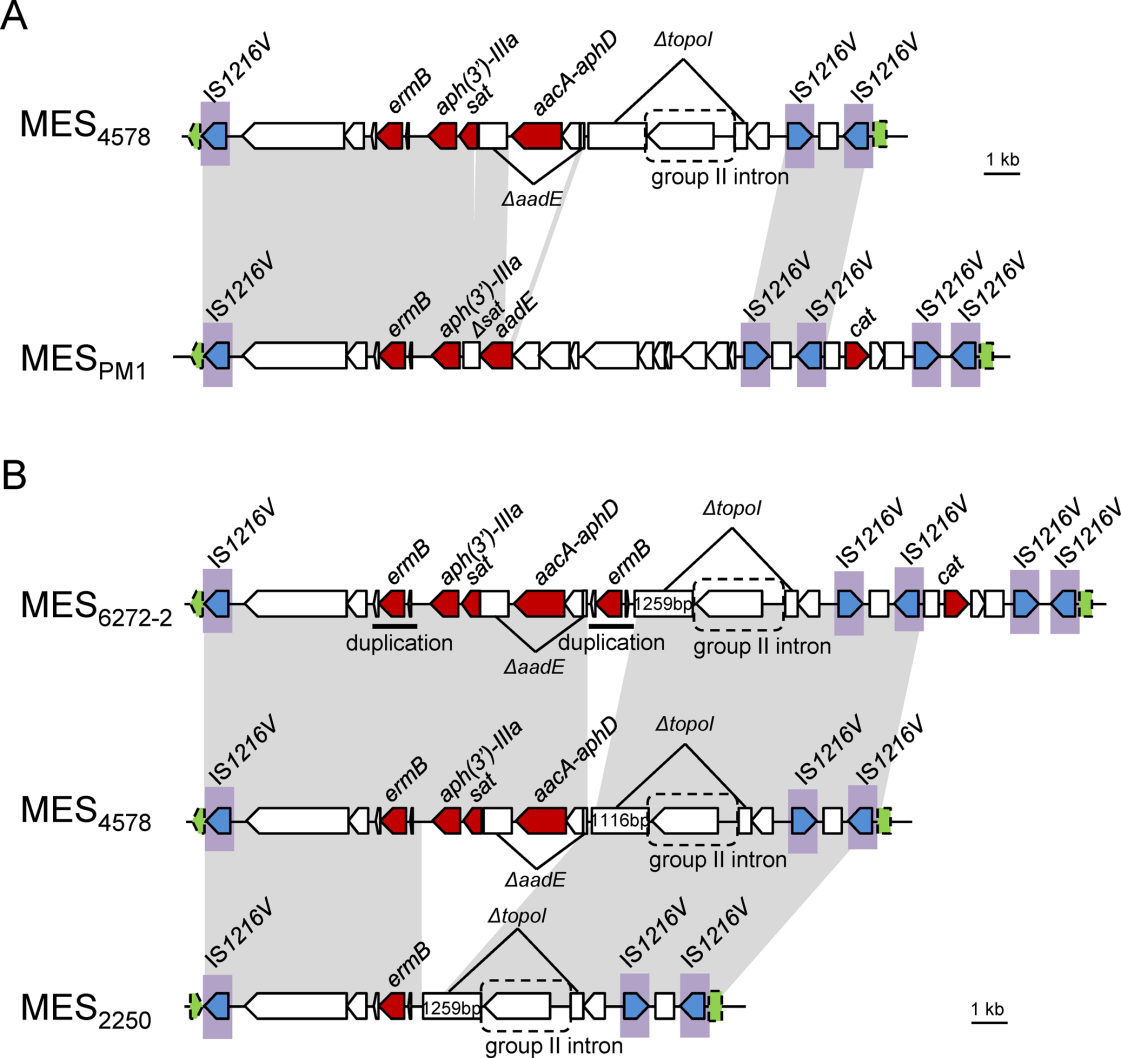
The comparison of MES structures integrated into the *sasK* gene. (A) MES_4578_ in ST59/SCC*mec* IVg MRSA strain 4578 is compared to MES_PM1_ in ST59/SCC*mec* V (5C2&5) MRSA strain PM1 (accession number AB699882) [23]. (B) MES_6272-2_, MES_4578_ and MES_2250_ in three ST59/SCC*mec* IVg MRSA strains 6272–2, 4578 and 2250, respectively, are compared. Homologous regions are shaded in gray. A Greek delta symbol indicates a truncated coding sequence. IS*1216V* with a transposase gene (*tnp*) is shaded in blue. The *sasK* gene is indicated in green arrow and box with dashed line.

**Fig 3:**
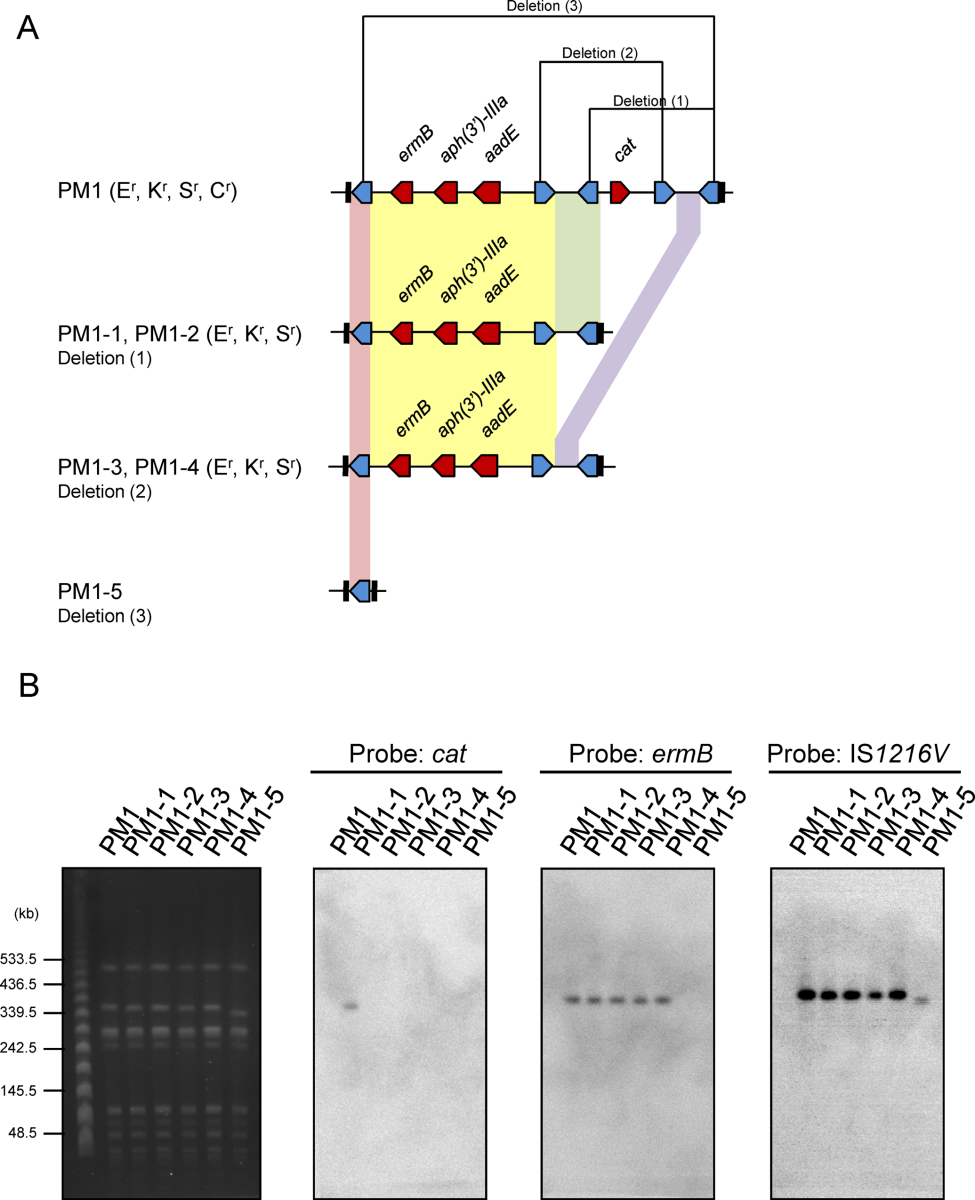
Selection for loss of antibiotic resistance in ST59/SCC*mec* V (5C2&5) MRSA strain PM1. (A) Cartoon representation of MES structures in PM1 and the generated colonies PM1-1 to PM1-5. (B) SmaI-digested PFGE of PM1 and PM1-1 to PM1-5 and the Southern blot. The SmaI-digested DNA separated by PFGE is shown in the left. The DNA was transferred to a nylon membrane and detected by Southern blot hybridization with DIG-labeled *cat*-, *ermB*- and IS*1216V*-specific probes, which are shown on the right. Abbreviations: E^r^, erythromycin resistant; K^r^, kanamycin resistant; S^r^, streptomycin resistant; C^r^, chloramphenicol resistant.

Sequence analysis revealed that MES_4578_ was flanked with enterococcal IS*1216V* and inserted into the 8-bp *att* duplication site within the *sasK* gene, similar to MES_PM1_ (Fig 2A). The left side of MES_4578_ contained an antibiotic resistance gene cluster *ermB*-(*aph(3')-IIIa*)-*sat*-*aadE-*(*aacA-aphD*), which is responsible for erythromycin/clindamycin, kanamycin, streptothricin, streptomycin and gentamicin resistance. The antibiotic resistance gene cluster was present in both MES_4578_ and MES_PM1_, albeit with some deletions or insertions (Fig 2A and S4 Table). The *sat* gene remained intact in MES_4578_, although it was truncated in MES_PM1_ due to a 62-bp deletion. In contrast, the *aadE* gene was intact in MES_PM1_ but was disrupted by the insertion of *aacA-aphD* and an ORF in MES_4578_, which explained the marked difference in the streptomycin and gentamicin resistance pattern in SCC*mec* IV MRSA and SCC*mec* V (5C2&5) MRSA. The region downstream of the antibiotic resistance gene cluster to the second IS*1216V* was quite different in MES_4578_ and MES_PM1_. The 3′ region of MES_4578_, between two IS*1216V* elements, showed 99.9% DNA sequence identity to that in MES_PM1_. However, the *cat* gene (responsible for chloramphenicol resistance) and the additional two IS*1216V* elements were lacking in MES_4578_. Our previous studies on clinical isolates revealed that excision/insertion event may take place between two direct repeats of IS*1216V*, leading to the distinct resistance patterns among the ST59/SCC*mec* V (5C2&5) clinical isolates [23]. In the present study, the excision/insertion event was further shown by selection for loss of antibiotic resistance in PM1, generating five strains with varied drug resistance patterns and MES structures (Fig 3A). PFGE separation of SmaI-digested genomic DNAs followed by Southern blot hybridization with the DIG-labeled *cat*-, *ermB*- and IS*1216V*-specific probes further confirmed the excision/insertion event in strain PM1 (Fig 3B).

The SCC*mec* IV MRSA showed more complex antimicrobial resistance patterns (S3 Table). Therefore, two SCC*mec* IV MRSA strains, 6272–2 (resistant to erythromycin, kanamycin, gentamicin and chloramphenicol) and 2250 (resistant to erythromycin only), displaying different antimicrobial resistance patterns were included to determine the full sequences of the antibiotic resistance elements. Sequence analysis revealed that the two elements, MES_6272-2_ and MES_2250_, were also flanked with enterococcal IS*1216V* elements and inserted into the *sasK* gene (Fig 2B). MES_6272-2_ had an additional *ermB* downstream of the *aacA-aphD* compared to MES_4578_. MES_2250_ lacked the antibiotic resistance gene cluster from *aph(3')-IIIa* to *aacA-aphD* and was therefore resistant to erythromycin only. Different lengths of the 5'-end-truncated topoisomerase I gene in MES_4578_ (1661 bp) and in MES_6272-2_ and MES_2250_ (1799 bp and 1800 bp) indicated that MES_2250_ and MES_4578_ may be generated from MES_6272-2_ independently. Moreover, MES_6272-2_ harbored the *cat* gene (chloramphenicol resistance), which was surrounded by four copies of IS*1216V*, displaying 99.2% DNA sequence similarity to that in MES_PM1_. The rightmost sides of the MES_4578_ and MES_2250_ sequences both contained two IS*1216V* elements and lacked the *cat* gene, suggesting that excision/insertion event also took place in SCC*mec* IV MRSA between two direct repeats of IS*1216V*, as we previously described in SCC*mec* V (5C2&5) MRSA. Detailed comparisons of ORFs in each MES are shown in S4 Table.

### The structure of νSAβ

**Fig 4:**
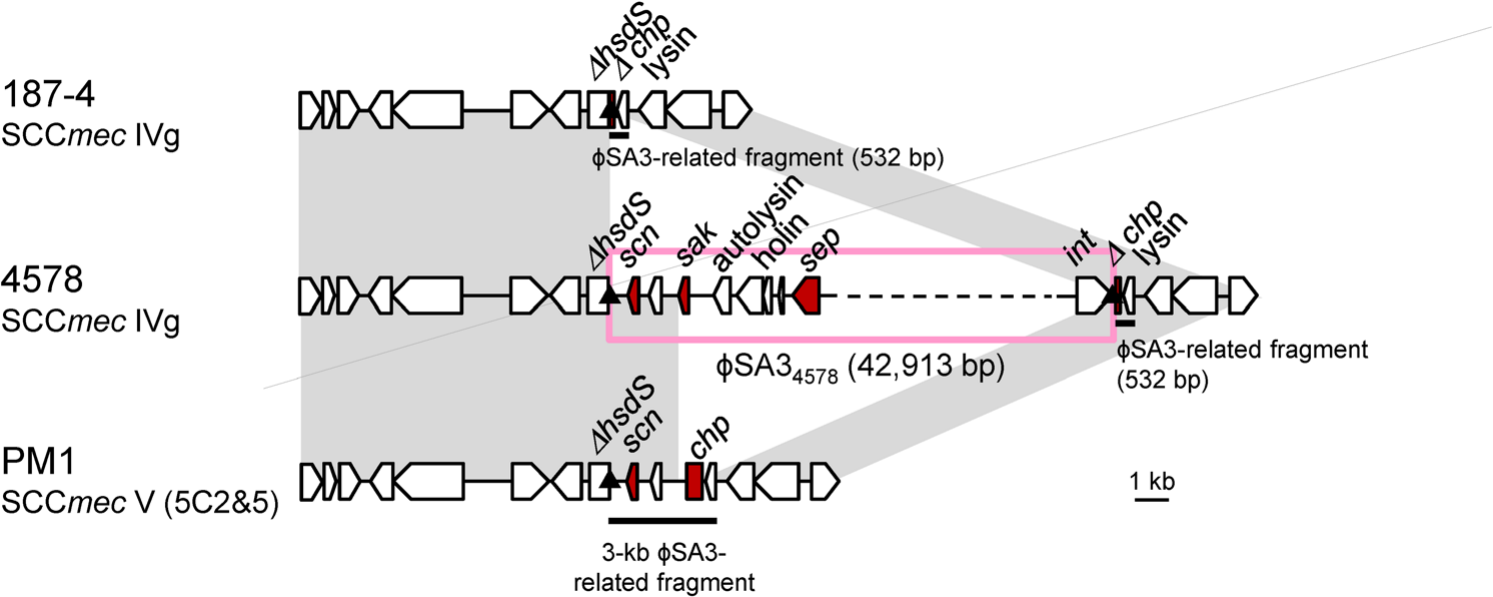
The νSaβ structure of strains 4578, PM1 and 187–4. The strains 4578 and 187–4 are ST59/SCC*mec* IVg MRSA carrying IEC type G or no IEC, respectively. Strain PM1 is ST59/SCC*mec* V (5C2&5) MRSA with IEC type C. Homologous regions are shaded. A Greek delta symbol indicates a truncated coding sequence. The *att* sequence is indicated by a triangle. Arrows with dashed lines indicate nearly complete coding sequence.

A comparison of the νSaβ structures in 4578 (SCC*mec* IV MRSA) and PM1 (SCC*mec* V (5C2&5) MRSA) is shown in Fig 4 and S5 Table. The two strains both carried truncated *hsdS* genes but harbored different IEC types. PM1 carried IEC type C (*chp* and *scn*), while 4578 carried IEC type G (*sak*, *scn* and *sep*). Remarkably, IEC type G in 4578 was carried by a 42.9-kb bacteriophage (φSA3_4578_). The φSA3_4578_, which belonged to the *Siphoviridae* family, was assigned to the φSA3 group based on the integrase gene sequence. As previously described, the φSA3 group is usually specifically integrated within the *hlb* gene and produces a 13-bp *att* sequence duplication at both ends [25]. However, φSA3_4578_ was translocated into νSaβ and was still demarcated by the same *att* sequences. Interestingly, the νSaβ in 4578 also harbored a 141-bp *chp* remnant and the lysin gene (φSA3-related fragment) just downstream of φSA3_4578_. This region (532 bp) showed 99.9% DNA sequence similarity to the corresponding region in PM1.

The IEC-lacking SCC*mec* IV MRSA strain 187–4 was subsequently used to analyze the genetic organization of νSaβ. As shown in Fig 4, the sequence of νSaβ in strain 187–4 was almost identical to the corresponding region in strain 4578. However, the φSA3 sequence was absent in strain 187–4, in accordance to its IEC-lacking genotype.

### Comparisons of mobile genetic elements among CC59 *S*. *aureus* strains

Based on the resolved sequences of the MES-related structures φSA3 and νSaβ, the 195 CC59 *S*. *aureus* strains were analyzed to determine the distribution of the mobile genetic elements. As shown in Table 2 and S3 Table, the multidrug-resistant structure MES_6272-2_ (gentamicin-resistant but streptomycin-susceptible) and the related structures MES_4578_ and MES_2250_ were found exclusively in SCC*mec* IV MRSA except for two MSSA strains. MES_PM1_ (streptomycin-resistant but gentamicin-susceptible) was mainly distributed in SCC*mec* V (5C2&5) MRSA and MSSA, although 22% of SCC*mec* IV MRSA strains carried MES_PM1_. The IS*1216V*/*cat*-related segregants, which could result from IS*1216V*-mediated excision/insertion in any MES and cause the loss of erythromycin, kanamycin, streptomycin and gentamicin resistance, were found to be evenly distributed in SCC*mec* IV MRSA, SCC*mec* V (5C2&5) MRSA or MSSA.

**Table 2:** Distribution of mobile genetic elements related to multidrug resistance or IEC.

	No. (%) of strains		
	SCC*mec* IV (n = 91)	SCC*mec* V (5C2&5) (n = 74)	MSSA (n = 30)
Antibiotic resistance elements or related structures inserted into *sasK* ^a^			
MES_6272-2_ (E^r^, K^r^, G^r^) ^b^	51 (56.7)	0 (0)	1 (3.3)
MES_4578_ (E^r^, K^r^, G^r^) ^b^	8 (8.8)	0 (0)	1 (3.3)
MES_2250_ (E^r^) ^b^	6 (6.6)	0 (0)	0 (0)
MES_PM1_ (E^r^, K^r^, S^r^) ^b^	20 (22.0)	66 (89.2)	25 (83.3)
IS*1216V* or *cat*-related segregants	5 (5.5)	8 (10.8)	3 (10)
Untypable	1 (1.1)	0 (0)	0 (0)
Elements harboring IEC			
φSA3 translocated into νSaβ:			
IEC type B	1 (1.1)	0 (0)	0 (0)
IEC type D	3 (3.3)	0 (0)	0 (0)
IEC type G	61 (67)	0 (0)	4 (13.3)
φSA3 integrated into *hlb*: IEC type B ^c^	11 (12.1)	0 (0)	0 (0)
φSA3-related fragment in νSaβ: IEC type C	8 (8.8)	74 (100)	25 (83.3)
Loss of IEC	4 (4.4)	0 (0)	0 (0)
Untypable	3 (3.3)	0 (0)	1 (3.3)

^a^ E^r^: erythromycin resistant; K^r^: kanamycin resistant; G^r^: gentamicin resistant; S^r^: streptomycin resistant.

^b^ Classification of MES types is based on sequences of the antibiotic resistance gene cluster; the *cat* gene and its surrounding regions are not included because they are universal among different MES types.

^c^ All the strains showed a "loss of IEC" pattern in νSaβ except strain 7576, which was untypable.

The νSaβ region with φSA3 translocation or with φSA3-related fragment was found in all of the CC59 *S*. *aureus* strains. For the distribution, SCC*mec* V (5C2&5) MRSA harbored φSA3-related fragment with IEC type C with no exception. In MSSA, the φSA3-related fragment with IEC type C was dominant (n = 25), followed by φSA3 translocation in νSaβ with IEC type G (n = 4). For SCC*mec* IV MRSA, although φSA3 translocation in νSaβ with IEC type G was frequently found in SCC*mec* IVg MRSA, the elements harboring IEC were quite different in the remaining SCC*mec* IV MRSA strains (see below). Eleven strains harbored φSA3 with IEC type B integrated into its regular site *hlb*. Within the 11 strains, 10 strains lacked any IEC in the νSaβ, similar to strain 187–4 (Fig 4), while one strain remained untypable. Eight strains acquired IEC type C via a φSA3-related fragment in νSaβ, similar to that in SCC*mec* V (5C2&5) MRSA. Four strains lacked any IEC in the entire genome. Three strains harbored IEC in the νSaβ with untypable structure and were without φSA3 integrated into *hlb*.

### Diversity of the SCC*mec* IV MRSA

To further understand the genetic relatedness of the 91 SCC*mec* IV MRSA strains, SCC*mec* IV subtypes were determined. As shown in Table 3, a total of 81 strains (89%) carried SCC*mec* IVg, in agreement with the genomic analysis of ST59/SCC*mec* IV MRSA type strain 4578, which showed 98.8% nucleotide sequence similarity to the SCC*mec* IVg sequence (accession number DQ106887). For the 10 non-subtype-g SCC*mec* IV strains, SCC*mec* IVa was the dominant subtype (n = 7), followed by SCC*mec* IV nontypable (n = 2) and SCC*mec* IVc (n = 1). The distributions of the virulence factor gene *luk*_*PV*_*SF*, the elements harboring IEC (φSA3-related fragment in νSaβ or φSA3 translocated into νSaβ) and the MES structures (MES_PM1_ or MES_6272-2_ and related elements) were quite different in non-subtype-g SCC*mec* IV MRSA and SCC*mec* IVg MRSA (Table 3). For the 10 strains with non-subtype-g SCC*mec* IV, the characteristics (presence of *luk*_*PV*_*SF* gene, φSA3-related fragment in νSaβ and the MES_PM1_ structures) were much more similar to SCC*mec* V (5C2&5) MRSA than to SCC*mec* IVg MRSA.

**Table 3:** Distribution of virulence genes or mobile genetic elements within SCC*mec* IV MRSA.

	No. (%) of strains	
	Non-subtype-g SCC*mec* IV ^a^ (n = 10)	SCC*mec* IVg (n = 81)
*luk*_*PV*_*SF* ^b^	8 (80)	4 (49.4)
Elements harboring IEC		
φSA3-related fragment in νSaβ ^b^	8 (80)	0 (0)
φSA3 integrated into *hlb*	1 (10)	10 (12.3)
φSA3 translocated into νSaβ ^b^	0 (0)	69 (85.2)
Untypable	1 (10)	2 (2.5)
Antibiotic resistance elements or related structures inserted into *sasK*		
MES_PM1_ ^b^	9 (90)	11 (13.6)
MES_6272-2_ and related elements ^b^	0 (0)	65 (80.2)
IS*1216V* or *cat*-related segregants	1 (10)	4 (4.9)

^a^ Subtypes of non-subtype-g SCC*mec* IV MRSA: SCC*mec* IVa (n = 7), SCC*mec* IVc (n = 1) and SCC*mec* IV nontypable (n = 2).

^b^ Statistically significant difference between non-subtype-g SCC*mec* IV and SCC*mec* IVg MRSA strains (*P* value <0.05 by Fisher’s exact test).

Considering the unique patterns of non-subtype-g SCC*mec* IV MRSA within the ST59/SCC*mec* IV MRSA, the PVL-positive ST59/SCC*mec* IVa MRSA strain USA1000 isolated from the United States was included for investigation. The 3-kb φSA3-related fragment was present in νSaβ of USA1000, similar to the structure in SCC*mec* V (5C2&5) MRSA. However, no IS*1216V* or MES-related structure was inserted into the *sasK* gene, which was quite different from the case for CC59 *S*. *aureus* strains isolated from Taiwan.

### Cluster analysis of CC59 *S*. *aureus* strains

**Fig 5:**
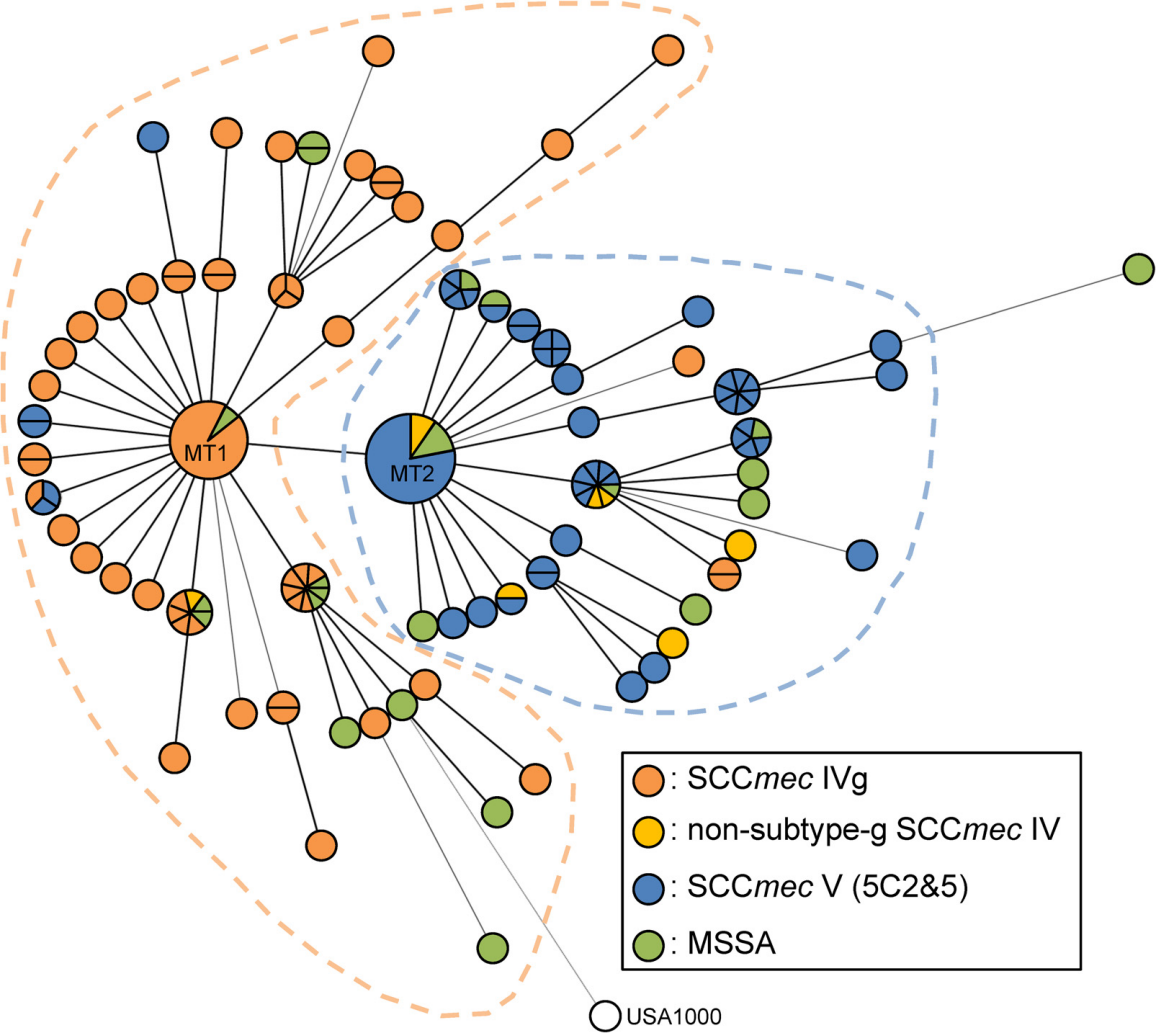
Minimum spanning tree of CC59 *S*. *aureus* strains constructed by MLVA. Each circle represents an MLVA type. Circles or sectors of circles with orange, yellow, blue or green colors denote SCC*mec* IVg MRSA, non-subtype-g SCC*mec* IV MRSA, SCC*mec* V (5C2&5) MRSA or MSSA, respectively. The USA1000, which is ST59 MRSA in the United States, is shown in white. The size of each circle is proportional to the number of strains.

A minimum spanning tree based on the MLVA-16_Orsay_ (with 16 loci) is shown in Fig 5. There were two dominant MLVA types, MT1 (MLVA profile: 7-7-2-2-2-2-2-4-2-3-1.5-0-3-10-16-3) and MT2 (MLVA profile: 7-7-2-2-2-2-2-4-2-3-1.5-0-3-7-16-3). The major difference between MT1 and MT2 was the repeat number in locus sa0964 (10 in MT1 and 7 in MT2); the repeat numbers in the other 15 loci were identical. MT1 and its adjacent neighbors formed a clade that was predominantly SCC*mec* IVg MRSA (orange dashed-line circle, Fig 5); MT2 and its relatives formed the other clade that mostly consisted of SCC*mec* V (5C2&5) MRSA and non-subtype-g SCC*mec* IV MRSA (blue dashed-line circle, Fig 5); MSSA was distributed in the both clades. In addition, the USA1000 was grouped with SCC*mec* IVg MRSA clade with a longer branch.

## Discussion

The CC59 *S*. *aureus* in Taiwan has been reported to have unique multidrug resistance mechanisms and νSaβ structures, which are distinct from those of other lineages of *S*. *aureus* [19, 23]. The present study, combining molecular characterization of the mobile genetic elements and phylogenetic analysis based on MLVA, further elucidated these differences, as follows: (i) genetic relatedness within the CC59/SCC*mec* IV MRSA strains was diverse, with the non-subtype-g SCC*mec* IV MRSA phylogenetically closer to CC59/SCC*mec* V (5C2&5) MRSA than to CC59/SCC*mec* IVg MRSA; and (ii) CC59 MSSA strains were phylogenetically clustered within the SCC*mec* IVg MRSA or SCC*mec* V (5C2&5) MRSA, sharing both of their features.

Among the mobile genetic elements that we analyzed, νSaβ containing a φSA3-related fragment or translocated φSA3 were found in all CC59 *S*. *aureus*, including the USA1000 strain isolated from the United States, which suggested that the common progenitor of CC59 *S*. *aureus* may have already undergone chromosomal rearrangements to obtain IEC genes in the νSaβ. Acquisition of νSaβ containing a φSA3-related fragment leading to disruption of type I restriction-modification system was found exclusively in CC59 *S*. *aureus*, especially dominant in SCC*mec* V (5C2&5) MRSA as we previously reported [23]. In the current study, another unique νSaβ structure harboring translocated φSA3 in CC59 *S*. *aureus*, especially in SCC*mec* IVg MRSA was found, which indicated that the above chromosomal rearrangement events in νSaβ are the key features to CC59 *S*. *aureus*.

As for the enterococcal IS*1216V*-mediated MES structures, MES_PM1_ and MES_6272-2_ and related elements were found in all CC59 *S*. *aureus* strains, but the MES type distribution was quite different among SCC*mec* IVg, non-subtype-g SCC*mec* IV and SCC*mec* V (5C2&5) MRSA (Tables 2 and 3). The 5′ region of MES_PM1_ was identical to the corresponding region of a plasmid (pLG2) of *Enterococcus faecalis* [23, 42], while the left side of MES_6272-2_ was highly similar to the IS*1216V*-*ermB*-(*aph(3')-IIIa*)-*sat*-*ΔaadE-*(*aacA-aphD*)-*ΔaadE* structure of *Enterococcus faecium* isolated in Taiwan (our unpublished results). These results suggest that two independent events of acquisition from enterococci led to the varied MES distributions.

**Fig 6:**
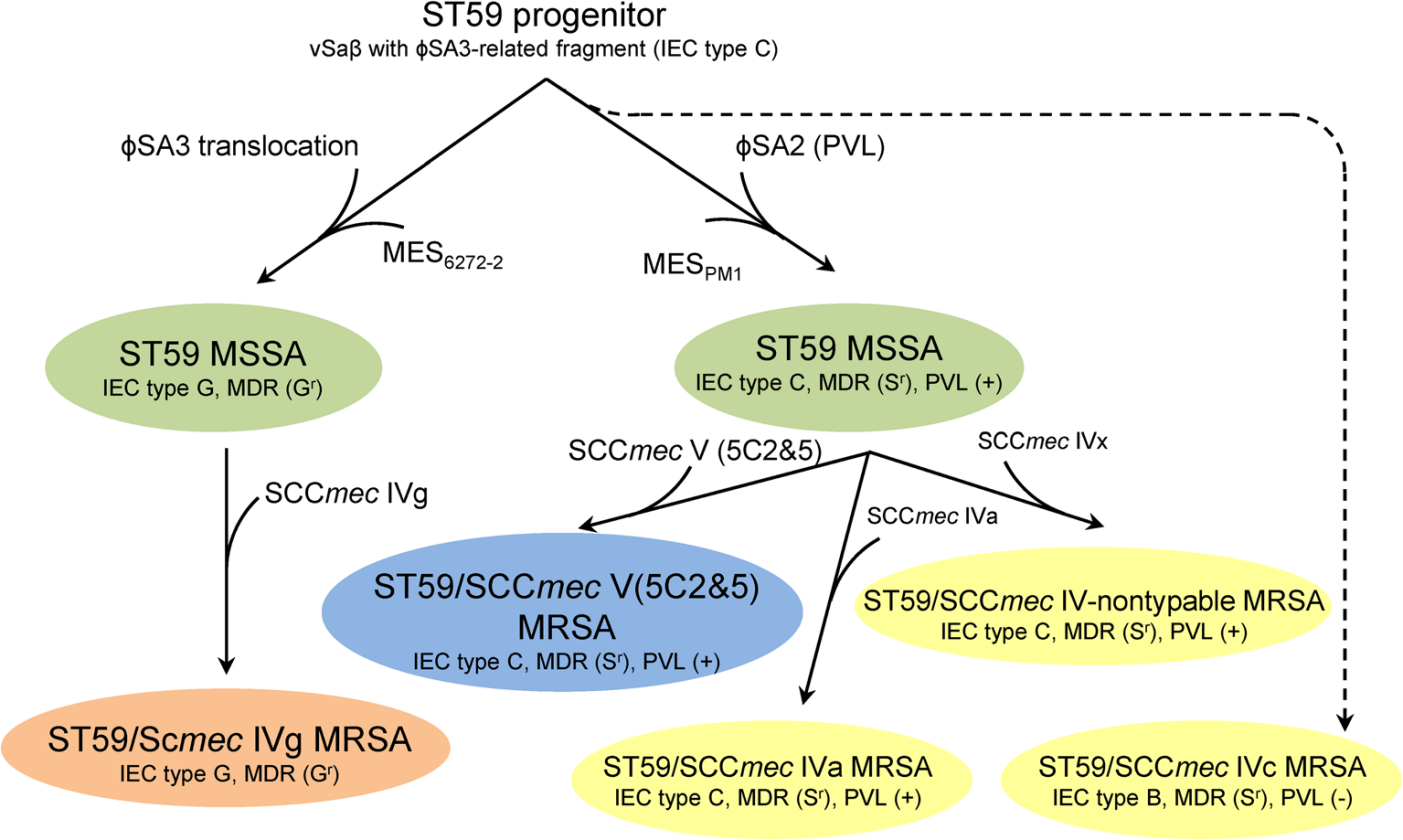
Proposed evolutionary history of CC59 *S*. *aureus* in Taiwan. SCC*mec* IVg MRSA, non-subtype-g SCC*mec* IV MRSA, SCC*mec* V (5C2&5) MRSA or MSSA with the characteristics found in this study are shaded in orange, yellow, blue or green, respectively. Dash line indicates an uncertain evolutionary route. MDR: multidrug resistance; S^r^: streptomycin resistance; G^r^: gentamicin resistance.

Based on the above findings, we propose a hypothetical ST59 MRSA evolutionary history in Taiwan (Fig 6). For the two major ST59 MRSA clones (SCC*mec* IVg and SCC*mec* V (5C2&5)), a common CC59 MSSA progenitor appeared first, and its νSaβ harbored a φSA3-related fragment with IEC type C. Next, two separate evolutionary routes were followed. In one route, strains acquired MES_PM1_ and the PVL-positive φSA2, and finally, CC59 MSSA (IEC type C, MES_PM1_ and PVL-positive) acquired SCC*mec* V (5C2&5) to become MRSA; in the other route, strains underwent φSA3 translocation and acquired MES_6272-2_, and finally, the MSSA strains (IEC type G, MES_6272-2_ and PVL-negative) acquired SCC*mec* IVg to become one of the dominant clones in CC59 MRSA. For the three minor ST59 MRSA clones characterized as SCC*mec* IVa, SCC*mec* IVc and SCC*mec* IV nontypable, they were phylogenetically related to SCC*mec* V (5C2&5) MRSA based on the minimum spanning tree constructed by MLVA (Fig 5). The acquisition events in SCC*mec* IVa and SCC*mec* IV nontypable MRSA were similar to that in SCC*mec* V (5C2&5) MRSA except for the last step to acquire SCC*mec* elements. In contrast, the SCC*mec* IVc MRSA strain was PVL-negative and was characterized as untypable νSaβ with IEC type B, indicating this strain underwent different chromosomal rearrangement events.

CC59 *S*. *aureus* is the predominant CA-MRSA clone in Asia, especially in Taiwan and China [10, 43]. Previous studies have indicated that the SCC*mec* V (5C2&5) sequences of CC59 MRSA in the two areas are divergent. In strains from Taiwan, SCC*mec* V (5C2&5) has two distinct *ccrC* genes (*ccrC1* allele 2 and allele 8), while strains isolated from China carry only one *ccrC* gene (SCC*mec* V (5C2)) [44, 45]. Our results further indicated that the genetic backgrounds of the major SCC*mec* IV MRSA strains circulating in Taiwan and China are also different. ST59/SCC*mec* IVa MRSA carrying the PVL gene at a high rate was found to be the dominant clone among the pediatric population in China [45, 46]. However, the major clone of the 91 SCC*mec* IV MRSA strains that we analyzed is PVL-negative SCC*mec* IVg MRSA; the isolation rate of PVL-positive SCC*mec* IVa MRSA was low (7.7%, 7/91) (Table 3).

MLVA is a permissive molecular typing tool with stronger phylogenetic value than MLST or *spa* typing [47]. However, only one locus can effectively discriminate ST59/SCC*mec* IVg MRSA and ST59/SCC*mec* V (5C2&5) MRSA within the 16 loci used in the present study. These results implied that the branching of ST59/SCC*mec* IVg MRSA and ST59/SCC*mec* V (5C2&5) MRSA may have happened recently. Further studies using whole genome sequencing combined with molecular clock analysis are needed to precisely elucidate the evolutionary history of CC59 *S*. *aureus*.

The IEC, which can counteract antibacterial activity of the human innate immune system [48, 49], is widely distributed among *S*. *aureus* strains of human origin [25, 50]. CC59 *S*. *aureus* has acquired IEC in the νSaβ without inactivation of *hlb*, ensuring beta-hemolysin production. Beta-hemolysin contributes to the first stages of nasal colonization, which was shown by comparing *S*. *aureus* NCTC 8325–4 strains with an intact *hlb* or a *hlb* disrupted by φSA3 [51, 52]. The spontaneous and precise excision of φSA3 in the CA-MRSA clone MW2 was shown to significantly increase its skin colonization ability [53]. Indeed, previous molecular epidemiology studies in Taiwan indicated ST59 clones accounted for >77% of MRSA isolates carried by healthy children and adolescents [54–56]. In addition, the φSA3 dynamics during infections that lead to complete restoration of beta-hemolysin production and promote host adaption and increase virulence have been reported [52, 53, 57]. Hence, spontaneously harboring IEC and intact *hlb* may increase fitness for colonization and infection in the majority (180/195) of CC59 *S*. *aureus*.

In conclusion, our analyses support a proposed depiction of the localized evolution of CC59 *S*. *aureus*. The acquisition of several mobile genetic elements increased antimicrobial resistance and adaptation, which has led CC59 *S*. *aureus* to become the most successful CA-MRSA clone in Taiwan.

## Supporting Information

S1 Fig Cartoon representation of PCR mapping. Schematic maps of (A) MES, (B) νSaβ and (C) φSA3 integrated within *hlb* are shown. The arrows below the structures indicate PCR primers, which are listed in S2 Table.
(PDF)

S1 Table Information of bacterial isolates. (PDF)

S2 Table Primers used in this study. (PDF)

S3 Table Antimicrobial resistance patterns in 195 CC59 *S*. *aureus* strains. (PDF)

S4 Table Comparison of genetic contents of MES structures in CC59 MRSA. (PDF)

S5 Table Comparison of genetic contents of νSaβ structures in CC59 MRSA. (PDF)
