# Supplementary material for: Molecular Evolutionary Pathways toward Two Successful Community-Associated but Multidrug-Resistant ST59 Methicillin-Resistant Staphylococcus aureus Lineages in Taiwan: Dynamic Modes of Mobile Genetic Element Salvages
Source: PLoS One. 2016 Sep 8;11(9):e0162526. doi: 10.1371/journal.pone.0162526 (PMC5015870; doi:10.1371/journal.pone.0162526)
Supplement: S1 Fig — Schematic maps of (A) MES, (B) νSaβ and (C) φSA3 integrated within hlb are shown. The arrows below the structures indicate PCR primers, which are listed in S2 Table. (PDF) [file pone.0162526.s001.pdf]

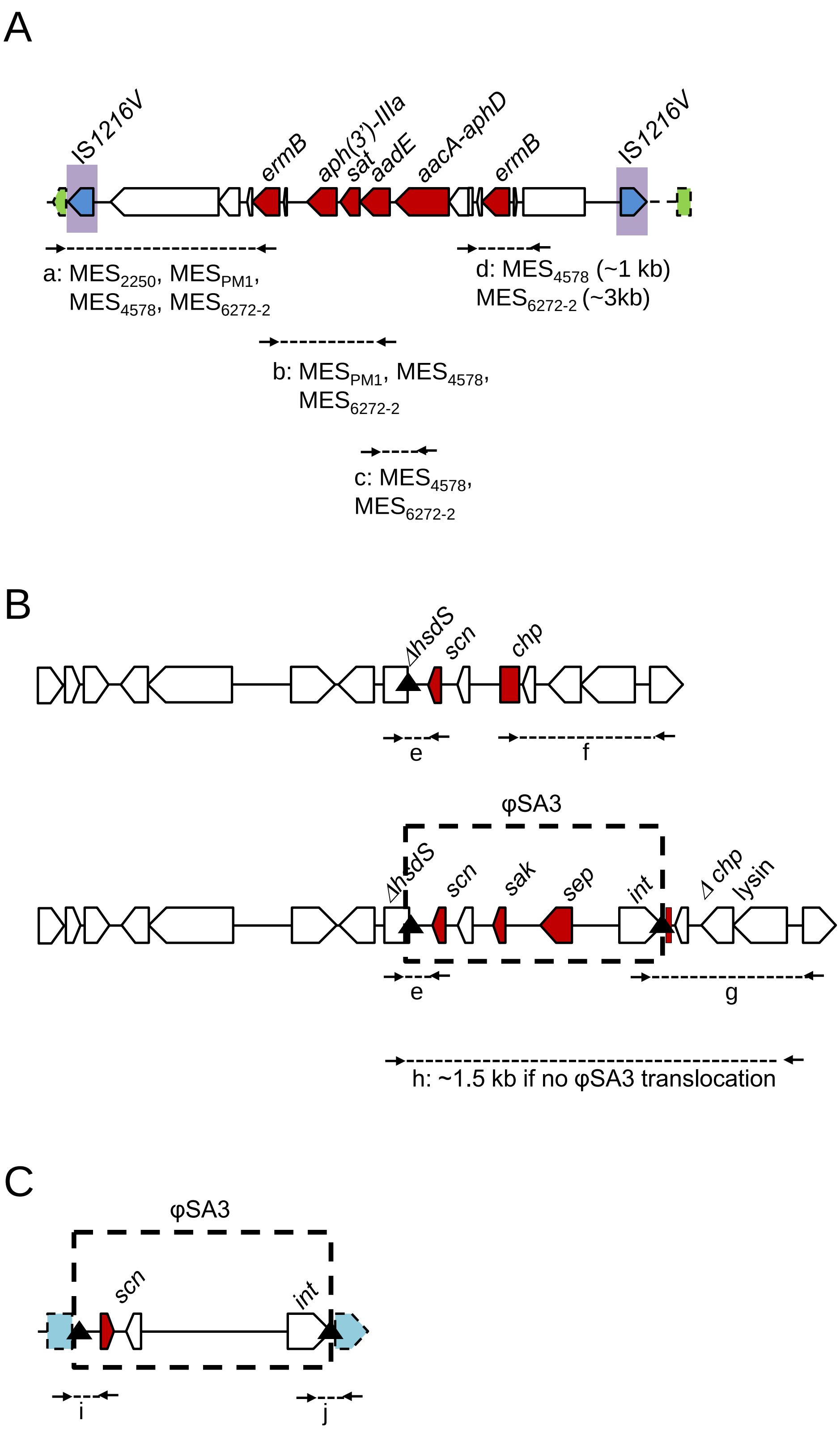

S1 Fig. Cartoon representation of PCR mapping.

Schematic maps of (A) MES, (B) νSaβ and (C) φSA3 integrated within *hlb* are shown. The arrows below the structures indicate PCR primers, which are listed in S2 Table.
