# Supplementary material for: Molecular Evolutionary Pathways toward Two Successful Community-Associated but Multidrug-Resistant ST59 Methicillin-Resistant Staphylococcus aureus Lineages in Taiwan: Dynamic Modes of Mobile Genetic Element Salvages
Source: PLoS One. 2016 Sep 8;11(9):e0162526. doi: 10.1371/journal.pone.0162526 (PMC5015870; doi:10.1371/journal.pone.0162526)
Supplement: S1 Table — (PDF) [file pone.0162526.s002.pdf]

S1 Table. Information of bacterial isolates.

Strain No.	Isolate date	ST type	*spa* type	SCC*mec*	Specimen	CA-MRSA[a]
1915	2000/01/19		t437	-	Blood	ND
4507	2000/02/12	59	t437	-	Blood	ND
135	2000/04/06		t7496	-	Blood	ND
2850	2000/04/29		t437	-	Blood	ND
3959	2000/05/08		t441	-	Blood	ND
6771	2000/08/24		t437	-	Blood	ND
248	2000/09/22		t437	-	Blood	ND
2575	2000/10/12		t437	-	Blood	ND
3809	2000/10/21		t441	-	Blood	ND
7259	2000/11/21		t437	-	Blood	ND
609	2005/1/2		t437	-	Blood	ND
3636	2005/5/16		t437	-	Blood	ND
7135	2005/7/28		t437	-	Blood	ND
3883	2005/9/9		t8886	-	Blood	ND
6414-1	2005/9/20		t437	-	Blood	ND
740	2005/10/16		t437	-	Blood	ND
6280	2005/11/20	59	t437	-	Blood	ND
8749	2005/12/6		t437	-	Blood	ND
288	2010/01/12		t437	-	Blood	ND
1642	2010/01/19	59	t437	-	Blood	ND
9930	2010/06/13		t437	-	Blood	ND
9145	2010/07/24		t3385	-	Blood	ND
413	2010/07/30		t437	-	Blood	ND
4235	2010/11/18		t437	-	Blood	ND
345	2010/12/17		t437	-	Blood	ND
7601	2011/3/7		t437	-	Blood	ND
2-1850	2011/4/24		t437	-	Blood	ND
5656	2011/10/11		t437	-	Blood	ND
8780	2011/12/13	59	t437	-	Blood	ND
1507	2011/12/26		t437	-	Blood	ND
1742	2000/1/17		t437	IVg	Blood	ND
3507	2000/2/2		t437	IVg	Blood	ND
6915	2000/3/6		t437	IVg	Blood	ND
8007	2000/3/16		t437	IVg	Blood	ND
8784	2000/3/23		t437	IVg	Blood	ND
396	2000/4/7		t437	IVa	Blood	ND
1929	2000/4/20		t437	IVg	Blood	ND
2351	2000/4/24	59	t437	IVa	Blood	ND
4287	2000/5/11	59	t3513	IVg	Blood	ND
4369	2000/5/12		t437	IVg	Blood	ND
6105	2000/5/26		t437	IVg	Blood	ND
7921	2000/6/12		t437	IVg	Blood	ND
9330	2000/6/22		t3485	IVg	Blood	ND
2697	2000/7/21		New type	IVg	Blood	ND
3706	2000/7/29		t437	IVg	Blood	ND
8526	2000/9/7		t437	IVg	Blood	ND
4456	2000/10/27		t437	IVg	Blood	ND
4572	2000/10/28		t437	IVg	Blood	ND
4773	2000/10/30		t437	IVg	Blood	ND
5324	2000/11/4		t437	IVg	Blood	ND

6940	2000/11/17		t437	IVg	Blood	ND
7061	2000/11/20		t437	IVg	Blood	ND
7169	2000/11/20		t437	IVg	Blood	ND
586	2005/1/2		t437	IVg	Blood	ND
7078	2005/2/11		t437	IVg	Blood	ND
244	2005/3/3		t437	IVg	Blood	ND
312	2005/3/3		t1751	IVg	Blood	ND
3850	2005/3/23		t441	IVg	Blood	ND
6578	2005/4/7		New type	IVg	Blood	ND
7666-1	2005/4/13		t437	IVg	Blood	ND
8196-1	2005/4/16		t437	IVg	Blood	ND
8844	2005/4/19		t437	IVg	Blood	ND
8993	2005/4/20		t437	IVg	Blood	ND
2994	2005/5/12		t437	IVg	Blood	ND
3943	2005/5/17	59	t437	IVg	Blood	ND
5594	2005/5/26		t441	IVg	Blood	ND
8401	2005/6/10		t437	IVg	Blood	ND
3401	2005/7/7		t437	IVg	Blood	ND
4533	2005/7/13		t437	IVg	Blood	ND
6083-2	2005/7/22		t437	IVg	Blood	ND
6385	2005/7/24		t437	IVg	Blood	ND
8061	2005/8/3		t437	IVg	Blood	ND
770	2005/8/19		t437	IVg	Blood	ND
807	2005/8/19		t437	IVg	Blood	ND
5514	2005/9/15		t441	IVg	Blood	ND
8483	2005/10/3	59	t437	SCC*mec* IV nontypable	Blood	ND
9772-1	2005/10/11		t437	IVg	Blood	ND
74	2005/10/12		t437	IVg	Blood	ND
929	2005/10/18		t437	SCC*mec* IV nontypable	Blood	ND
2370	2005/10/27		t437	IVg	Blood	ND
c8032	2005/11/4		t441	IVg	Blood	ND
4265	2005/11/9		t437	IVg	Blood	ND
4703	2005/11/10		t437	IVg	Blood	ND
5439	2005/11/14		t437	IVg	Blood	ND
5547	2005/11/15		t437	IVa	Blood	ND
1436	2005/12/24		t3513	IVg	Blood	ND
1476	2005/12/25		t437	IVg	Blood	ND
4136	2005/2/26	59	t437	IVg	sputum	Yes
4939	2005/3/2		t3485	IVg	surgical wound	Yes
6615	2005/3/9		t1751	IVg	pus	Yes
4516	2005/5/15	59	t437	IVg	sputum	Yes
5005	2005/5/17	59	t1751	IVg	umbilical discharge	Yes
5704	2005/5/19		t3424	IVg	pus	Yes
748	2005/6/7		t437	IVc	sputum	Yes
1576	2005/6/10		t437	IVg	eye	Yes
187-4	2010/01/11		t437	IVg	Blood	ND
1070	2010/01/16		t1751	IVg	Blood	ND
1265	2010/01/17		t437	IVg	Blood	ND
7576	2010/02/21		t437	IVa	Blood	ND
9852	2010/03/05		t437	IVg	Blood	ND
2413	2010/03/08		t437	IVg	Blood	ND
2250	2010/03/18		t437	IVg	Blood	ND
2738	2010/03/20		t437	IVg	Blood	ND

6272-2	2010/04/07	59	t437	IVg	Blood	ND
4578	2010/05/18	59	t437	IVg	Blood	ND
4830	2010/05/19		t4134	IVg	Blood	ND
8220	2010/06/04		t437	IVg	Blood	ND
8256	2010/06/04		t437	IVg	Blood	ND
6591	2010/07/12		t437	IVg	Blood	ND
8283	2010/07/20		t437	IVg	Blood	ND
6306	2010/10/12		t437	IVa	Blood	ND
6859	2010/10/14		t441	IVg	Blood	ND
2362	2010/10/31		t437	IVa	Blood	ND
2522	2010/11/10		t1950	IVg	Blood	ND
9391	2010/12/13		t437	IVg	Blood	ND
1161	2011/04/14		t437	IVg	Blood	ND
7649	2011/04/22		t437	IVg	Blood	ND
6077	2011/07/15		t437	IVa	Blood	ND
3768	2011/10/01	59	t437	IVg	Blood	ND
3858	2011/10/02	59	t437	IVg	Blood	ND
7261	2011/12/06		t437	IVg	Blood	ND
5401	2000/2/21		t437	SCC*mec* V (5C2&5)	Blood	ND
5819	2000/2/24		t437	SCC*mec* V (5C2&5)	Blood	ND
5923	2000/2/25		t437	SCC*mec* V (5C2&5)	Blood	ND
8005	2000/3/16		t437	SCC*mec* V (5C2&5)	Blood	ND
1136	2000/4/13	59	t437	SCC*mec* V (5C2&5)	Blood	ND
7416	2000/6/7		t437	SCC*mec* V (5C2&5)	Blood	ND
7448	2000/6/7		t437	SCC*mec* V (5C2&5)	Blood	ND
7860	2000/6/10		t437	SCC*mec* V (5C2&5)	Blood	ND
my7965	2000/7/7		t3485	SCC*mec* V (5C2&5)	Blood	ND
1199	2000/7/7		New type	SCC*mec* V (5C2&5)	Blood	ND
c4736	2000/7/18		t437	SCC*mec* V (5C2&5)	Blood	ND
2157	2000/10/9	59	t437	SCC*mec* V (5C2&5)	Blood	ND
5179-1	2000/11/2		t437	SCC*mec* V (5C2&5)	Blood	ND
6787-2	2000/11/16		t437	SCC*mec* V (5C2&5)	Blood	ND
7179	2000/11/20		t437	SCC*mec* V (5C2&5)	Blood	ND
8981	2000/12/5		t437	SCC*mec* V (5C2&5)	Blood	ND
9209	2000/12/7		t437	SCC*mec* V (5C2&5)	Blood	ND
104	2000/12/12		t437	SCC*mec* V (5C2&5)	Blood	ND
175	2000/12/13		t437	SCC*mec* V (5C2&5)	Blood	ND
355	2000/12/14		t437	SCC*mec* V (5C2&5)	Blood	ND
4526	2005/1/27		t437	SCC*mec* V (5C2&5)	Blood	ND
7322	2005/2/4		t437	SCC*mec* V (5C2&5)	Blood	ND
6092	2005/2/5		t437	SCC*mec* V (5C2&5)	Blood	ND
6534	2005/2/7		t441	SCC*mec* V (5C2&5)	Blood	ND
1432	2005/5/3		t437	SCC*mec* V (5C2&5)	Blood	ND
7796-2	2005/6/7		t437	SCC*mec* V (5C2&5)	Blood	ND
9124	2005/6/14		t437	SCC*mec* V (5C2&5)	Blood	ND
9648	2005/6/17	59	t437	SCC*mec* V (5C2&5)	Blood	ND
6415	2005/7/24		t1751	SCC*mec* V (5C2&5)	Blood	ND
7667	2005/8/1		t437	SCC*mec* V (5C2&5)	Blood	ND
3243	2005/9/3		t437	SCC*mec* V (5C2&5)	Blood	ND
6974	2005/9/23		t437	SCC*mec* V (5C2&5)	Blood	ND
7180	2005/9/25		t437	SCC*mec* V (5C2&5)	Blood	ND
7553	2005/9/27		t437	SCC*mec* V (5C2&5)	Blood	ND
8519	2005/10/3		t437	SCC*mec* V (5C2&5)	Blood	ND

8267	2005/12/2		t437	SCC*mec* V (5C2&5)	Blood	ND
8483	2005/12/4		t7501	SCC*mec* V (5C2&5)	Blood	ND
8753	2005/12/6	59	t437	SCC*mec* V (5C2&5)	Blood	ND
9039	2005/12/8		t437	SCC*mec* V (5C2&5)	Blood	ND
1035	2005/12/21		t437	SCC*mec* V (5C2&5)	Blood	ND
1262	2005/12/22		t437	SCC*mec* V (5C2&5)	Blood	ND
1686	2005/12/25		t437	SCC*mec* V (5C2&5)	Blood	ND
2803	2005/2/21		t437	SCC*mec* V (5C2&5)	eye	Yes
4010	2005/2/25		t437	SCC*mec* V (5C2&5)	external ear	Yes
5185	2005/3/3		t437	SCC*mec* V (5C2&5)	pus	Yes
5982	2005/3/7	59	t437	SCC*mec* V (5C2&5)	pus	Yes
6530	2005/3/8		t437	SCC*mec* V (5C2&5)	surgical wound	Yes
2841	2005/5/9	59	t437	SCC*mec* V (5C2&5)	pus	Yes
3165	2005/5/10		t437	SCC*mec* V (5C2&5)	skin pus	Yes
3242	2005/5/10		t437	SCC*mec* V (5C2&5)	discharge	Yes
7258	2005/5/25		t437	SCC*mec* V (5C2&5)	skin pus	Yes
8917-2	2005/6/1	59	t437	SCC*mec* V (5C2&5)	sputum	Yes
2073	2005/6/12		t437	SCC*mec* V (5C2&5)	abscess	Yes
9442	2010/01/07		t4145	SCC*mec* V (5C2&5)	Blood	ND
5312	2010/01/11		t437	SCC*mec* V (5C2&5)	Blood	ND
2082	2010/01/21	59	t437	SCC*mec* V (5C2&5)	Blood	ND
2910	2010/01/25		t437	SCC*mec* V (5C2&5)	Blood	ND
4076	2010/02/01	59		SCC*mec* V (5C2&5)	Blood	ND
6602	2010/02/15		t437	SCC*mec* V (5C2&5)	Blood	ND
5327	2010/04/02		t437	SCC*mec* V (5C2&5)	Blood	ND
9572	2010/07/26		t441	SCC*mec* V (5C2&5)	Blood	ND
2750	2010/08/22		t437	SCC*mec* V (5C2&5)	Blood	ND
9697	2010/09/11		t437	SCC*mec* V (5C2&5)	Blood	ND
3798	2010/09/30		t437	SCC*mec* V (5C2&5)	Blood	ND
9464	2010/10/26		t437	SCC*mec* V (5C2&5)	Blood	ND
1804	2010/12/24		t437	SCC*mec* V (5C2&5)	Blood	ND
414	2011/02/02	59	t437	SCC*mec* V (5C2&5)	Blood	ND
6395	2011/03/01		t437	SCC*mec* V (5C2&5)	Blood	ND
9717	2011/05/03		t437	SCC*mec* V (5C2&5)	Blood	ND
2400	2011/06/28		t437	SCC*mec* V (5C2&5)	Blood	ND
2409	2011/06/28		t437	SCC*mec* V (5C2&5)	Blood	ND
2615	2011/06/29		t437	SCC*mec* V (5C2&5)	Blood	ND
4343	2011/08/22		t437	SCC*mec* V (5C2&5)	Blood	ND
7889	2011/09/04		t437	SCC*mec* V (5C2&5)	Blood	ND

[a] ND, not determined
