# Supplementary material for: Molecular Evolutionary Pathways toward Two Successful Community-Associated but Multidrug-Resistant ST59 Methicillin-Resistant Staphylococcus aureus Lineages in Taiwan: Dynamic Modes of Mobile Genetic Element Salvages
Source: PLoS One. 2016 Sep 8;11(9):e0162526. doi: 10.1371/journal.pone.0162526 (PMC5015870; doi:10.1371/journal.pone.0162526)
Supplement: S2 Table — (PDF) [file pone.0162526.s003.pdf]

S2 Table. Primers used in this study.

Primer set	Primer name	Seqeunce (5'-3')
Detection of MES structure		
a	11F2	AGGCGAAACATTGGAAGT
	ermBR	GCAATGAAACACGCC
b	ermB	AGTAACGGTACTTAAATTGTTTAC
	aadE	ACTGGCTTAATCAATTTGGG
c	aadE-R	GTTCCCGCCTCTCTTCTA
	aacA-aphD-F	GAAGTACGCAGAAGAGA
d	aacA-aphD-R2	CATCTTCCCAAGGCTCTG
	4578R	AACGAGTGCTGAAATGAC
Detection of νSaβ structure		
e	beta-F4	CTGTTCTAACTTATTGGGCA
	scn-1	AGCACAAGCTTGCCAACATCG
f	chp-1	TTTACTTTTGAACCGTTTCCTAC
	beta-R3	GAGCGATTGTTCCATCT
g	phage3-intR	GCTTTGAAATCAGCCTGTAGAG
	beta island-R3	GAGCGATTGTTCCATCT
h	beta island-F4	CTGTTCTAACTTATTGGGCA
	IS232-F3	TTGGAAGTGAAGATGCCA
Detection of φSA3 within *hlb*		
i	hlb-1	GTTGGTGCTCTTACTGACAA
	scn-1	AGCACAAGCTTGCCAACATCG
j	hlb-2	TGTGTACCGATAACGTGAAC
	phage3-intR	GCTTTGAAATCAGCCTGTAGAG
