# Supplementary material for: Molecular Evolutionary Pathways toward Two Successful Community-Associated but Multidrug-Resistant ST59 Methicillin-Resistant Staphylococcus aureus Lineages in Taiwan: Dynamic Modes of Mobile Genetic Element Salvages
Source: PLoS One. 2016 Sep 8;11(9):e0162526. doi: 10.1371/journal.pone.0162526 (PMC5015870; doi:10.1371/journal.pone.0162526)
Supplement: S3 Table — (PDF) [file pone.0162526.s004.pdf]

S3 Table. Antimicrobial resistance patterns in 195 ST59 *S. aureus* strains.

Antimicrobial resistance patterns[a]	No. (%) of strains			Antibiotic resistance elements (n)
	SCC*mec* IV (n=91)	SCC*mec* V (5C2&5) (n=74)	MSSA (n=30)	
E, K, G, C	39 (42.9)	0 (0)	1 (3.3)	$MES_{6272\text{-}2}$ (50), MES_{4578} (9)
E, K, G	18 (19.8)	0 (0)	1 (3.3)	
E, K, S, C	12 (13.2)	42 (56)	15 (50)	MES_{PM1} (107)
E, K, S	7 (7.7)	22 (29.7)	9 (30)	
E, C	4 (4.4)	0 (0)	0 (0)	MES_{2250} (6)
E	2 (2.2)	0 (0)	0 (0)	
E, K, G, S, C	1 (1.1)	2 (2.7)	0 (0)	For gentamicin resistance: Tn*4001* (2), unknown (2); and for other antibiotic resistances: MES_{PM1} (4)
E, K, G, S	0 (0)	0 (0)	1 (3.3)	
E, K, C	2 (2.2)	0 (0)	0 (0)	$MES_{6272\text{-}2}$ (2)[b], untypable (1)[c]
E, K	1 (1.1)	0 (0)	0 (0)	
C	3 (3.3)	2 (2.7)	0 (0)	IS*1216V* (12), *cat*-related segregants (5)
Susceptible to the above antimicribials	3 (3.3)	6 (8)	3 (10)	

[a] Antimicrobials: E, erythromycin; K, kanamycin; G, gentamicin; S, streptomycin; C, chloramphenicol.

[b] A frame-shift mutation was found in the *aacA-aphD* gene (responsible for gentamicin resistance).

[c] The *aacA-aphD* gene is intact.
