# Supplementary material for: Molecular Evolutionary Pathways toward Two Successful Community-Associated but Multidrug-Resistant ST59 Methicillin-Resistant Staphylococcus aureus Lineages in Taiwan: Dynamic Modes of Mobile Genetic Element Salvages
Source: PLoS One. 2016 Sep 8;11(9):e0162526. doi: 10.1371/journal.pone.0162526 (PMC5015870; doi:10.1371/journal.pone.0162526)
Supplement: S4 Table — (PDF) [file pone.0162526.s005.pdf]

S4 Table. Comparison of genetic contents of MES structures in ST59 MRSA.[a]

Annotation or function in $MES_{6272\text{-}2}$	MES_{4578}	MES_{2250}	MES_{PM1}	May originate in enterococci
Transposase of IS*1216V*	+	+	+	Yes
Transposase of Tn*551*	+	+	+	Yes
Resolvase	+	+	+	Yes
Hypothetical protein	+	+	+	Yes
Erythromycin resistance transferase	+	+	+	Yes
Leader peptide	+	+	+	Yes
Aminoglycoside 3'-phosphotransferase	+	-	+	Yes
Streptothrin acetyletransferase	+	-	+/- (62-bp deletion)	Yes
streptomycin adenylyltransferase, 5'-end lacked	+	-	+ (intact)	Yes
Bifunctional aminoglycoside modifying enzyme	+	-	-	Yes
GNAT family acetyltransferase	+	-	-	Yes
streptomycin adenylyltransferase, 3'-end lacked	+	-	+ (intact)	Yes
Hypothetical protein	-	-	-	Yes
Erythromycin resistance transferase	-	-	-	Yes
Leader peptide	-	-	-	Yes
Type I topoisomerase, 5'-end lacked	+/- (143 bp deleted, 3'-end)	+	-	Yes
Putative group II intron reverse transcriptase	+	+	-	Yes
Type I topoisomerase, 3'-end lacked	+	+	-	Yes
Putative resolvase	+	+	-	Yes
Transposase of IS*1216V*	+	+	+	Yes
Replication initiation gene, 3'-end truncated	+	+	+	No
Transposase of IS*1216V*	+	+	+	Yes
replication initiation gene, 5'-end lacked	-	-	+	No
Chloramphenicol acetyltransferase	-	-	+	No
Relaxase accessory protein	-	-	+	No
Relaxase, truncated	-	-	+	No
Transposase of IS*1216V*	-	-	+	Yes
Transposase of IS*1216V*	-	-	+	Yes

[a] +: presence; -: absence; +/-: divergency
