# Supplementary material for: Molecular Evolutionary Pathways toward Two Successful Community-Associated but Multidrug-Resistant ST59 Methicillin-Resistant Staphylococcus aureus Lineages in Taiwan: Dynamic Modes of Mobile Genetic Element Salvages
Source: PLoS One. 2016 Sep 8;11(9):e0162526. doi: 10.1371/journal.pone.0162526 (PMC5015870; doi:10.1371/journal.pone.0162526)
Supplement: S5 Table — (PDF) [file pone.0162526.s006.pdf]

S5 Table. Comparison of genetic contents of νSaβ structures in ST59 MRSA.[a]

νSaβ in strain 4578: annotation or function	νSaβ in strain PM1	νSaβ in strain 187-4
orf1	+	+
orf2	+	+
orf3	+	+
hypothetical protein	+	+
putative helicase	+	+
hyaluronate lyase precusor	+	+
peptidase family	+	+
truncated HsdS	+	+
att site	+	+
staphylococcal complement inhibitor SCIN	+	-
phage lysin, N-acetylmuramoyl-L-alanine amidase, truncated	+	-
staphylokinase SAK	-	-
autolysin	-	-
holin	-	-
hypothetical protein	-	-
staplylococcal enterotoxin P, SEP	-	-
hypothetical protein	-	-
hypothetical protein	-	-
phi77 ORF044-like protein	-	-
phi77 ORF109-like protein	-	-
phi77 ORF002-like protein, phage minor structural protein	-	-
phi77 ORF004-like protein, putative phage tail component	-	-
phi77 ORF001-like protein, phage tail tape measure protein	-	-
phi77 ORF100-like protein	-	-
conserved hypothetical phage protein	-	-
hypothetical phage protein	-	-
phi77 ORF020-like protein, phage major tail protein	-	-
conserved hypothetical phage protein	-	-
conserved hypothetical phage protein	-	-
hypothetical protein	-	-
phi77 ORF006-like protein, putative capsid protein	-	-
phi77 ORF015-like protein, putative protease	-	-
phage portal protein	-	-
phi77 ORF003-like protein, phage terminase, large subunit	-	-
conserved hypothetical phage protein	-	-
phi77 ORF040-like protein	-	-
phi77 ORF026-like protein, putative phage transcriptional acti	-	-
phi77 ORF071-like protein	-	-
phiPVL ORF057-like protein, transcriptional activator RinB	-	-
phi77 ORF031-like protein	-	-
phi77 ORF069-like protein	-	-
hypothetical protein	-	-
putative dUTPase	-	-
hypothetical protein	-	-
phage protein	-	-
phiPVL ORF051-like protein	-	-
phiPVL ORF050-like protein	-	-
putative endodeoxyribonuclease	-	-
conserved hypothetical phage protein	-	-
phiPVL ORF046-like protein	-	-
single-strand binding protein	-	-
phiPVL ORF044-like protein	-	-
putative phage-related DNA recombination protein	-	-
phiPVL ORF41-like protein	-	-
phiPVL ORF39-like protein	-	-
conserved hypothetical phage protein	-	-
conserved hypothetical phage protein	-	-
hypothetical protein	-	-
hypothetical protein	-	-
phi77 ORF014-like protein, phage anti-repressor protein	-	-
conserved hypothetical phage protein	-	-
DNA-binding protein	-	-
phi77 ORF011-like protein, phage transcriptional repressor	-	-
putative exonuclease	-	-
hypothetical protein	-	-
hypothetical protein	-	-
integrase	-	-
att site	-	-
truncated chemotaxis inhibitory protein CHIPS, lack 5'-end	+/- (full length of CHIPS gene)	+
Phage lysin, N-acetylmuramoyl-L-alanine amidase, truncated	+	+
IstB helper protein	+	+
trasposase of IS*232*	+	+
Hypothetical protein	+	+

[a] +: presence; -: absence; +/-: divergency
